# miRNA-Mediated Interactions in and between Plants and Insects

**DOI:** 10.3390/ijms19103239

**Published:** 2018-10-19

**Authors:** Chade Li, Annette Y. P. Wong, Shuang Wang, Qi Jia, Wen-Po Chuang, William G. Bendena, Stephen S. Tobe, Seung Hwan Yang, Gyuhwa Chung, Ting-Fung Chan, Hon-Ming Lam, Jacqueline C. Bede, Jerome H. L. Hui

**Affiliations:** 1 State Key Laboratory of Agrobiotechnology, Centre of Soybean Research, School of Life Sciences, The Chinese University of Hong Kong, Hong Kong, China; R21381184@link.cuhk.edu.hk (C.L.); annettewongyp@gmail.com (A.Y.P.W.); tf.chan@cuhk.edu.hk (T.-F.C.); honming@cuhk.edu.hk (H.-M.L.); 2 Key Laboratory of Soil Environment and Plant Nutrition of Heilongjiang Province, Institute of Soil Fertilizer and Environment Resources, Heilongjiang Academy of Agricultural Sciences, Harbin 150086, China; wangshuang0726@163.com; 3 Key Laboratory for Genetics Breeding and Multiple Utilization of Crops, Ministry of Education/College of Crop Science, Fujian Agriculture and Forestry University, Fuzhou 350002, China; jiaqi@fafu.edu.cn; 4 Department of Agronomy, National Taiwan University, Taipei 10617, Taiwan; wenpo@ntu.edu.tw; 5 Department of Biology, Queen’s University, Kingston, ON K7L 3N6, Canada; bendenaw@queensu.ca; 6 Department of Cell and Systems Biology, University of Toronto, Toronto, ON M5S 3G5, Canada; stephen.tobe@utoronto.ca; 7 Department of Biotechnology, Chonnam National University, Yeosu 59626, Korea; ymichigan@jnu.ac.kr (S.H.Y.); chung@chonnam.ac.kr (G.C.); 8 Department of Plant Science, McGill University, 21,111 Lakeshore, Ste-Anne-de-Bellevue, Montreal, QC H9X 3V9, Canada; jacquie.bede@mcgill.ca

**Keywords:** microRNA, plant, insect

## Abstract

Our understanding of microRNA (miRNA) regulation of gene expression and protein translation, as a critical area of cellular regulation, has blossomed in the last two decades. Recently, it has become apparent that in plant-insect interactions, both plants and insects use miRNAs to regulate their biological processes, as well as co-opting each others’ miRNA systems. In this review article, we discuss the current paradigms of miRNA-mediated cellular regulation and provide examples of plant-insect interactions that utilize this regulation. Lastly, we discuss the potential biotechnological applications of utilizing miRNAs in agriculture.

## 1. MicroRNA (miRNA) Biogenesis and Regulation of Cellular Processes

Different classes of noncoding RNAs (ncRNAs) have been identified from animals, plants, and microorganisms. These ncRNAs are mainly classified based on their length: small ncRNA (18–30 nucleotide (nt)), medium ncRNA (31–200 nt), and long ncRNA (>200 nt) [1]. As they coordinate the regulation of key biological processes, understanding the roles of these ncRNAs and using their information against therapeutic or biotechnological targets has become an exciting research area in medicine and agriculture [2,3,4].

MiRNAs were first characterized from the nematode *Caenorhabditis elegans* in 1993. They were originally considered as non-protein coding RNA (lin-4) that regulated messenger RNA (lin-14) translation through base-pairing complementarity [5]. It is now clear that lin-4, as well as other miRNAs, function as post-transcriptional regulators via complementary base-pairing mainly at the 3’ untranslated region of mRNAs [5,6]). Loss-of-function studies have demonstrated that disruption of miRNA genes may result in serious regulatory defects, in turn determining developmental defects and several diseases [7]. In addition to the better-known miRNAs and piwi-interacting RNAs (piRNA), small RNAs include other members, such as ta-siRNA, nat-siRNA and ra-siRNA/ca-siRNA. These are generated from *TAS* genes, natural antisense transcripts and repeat-associated sequences, respectively [8,9]. In this review, we will focus on miRNA regulation of cellular mechanisms in plants and insects and then delve into examples of cross-organism manipulation by miRNAs.

**Figure 1:**
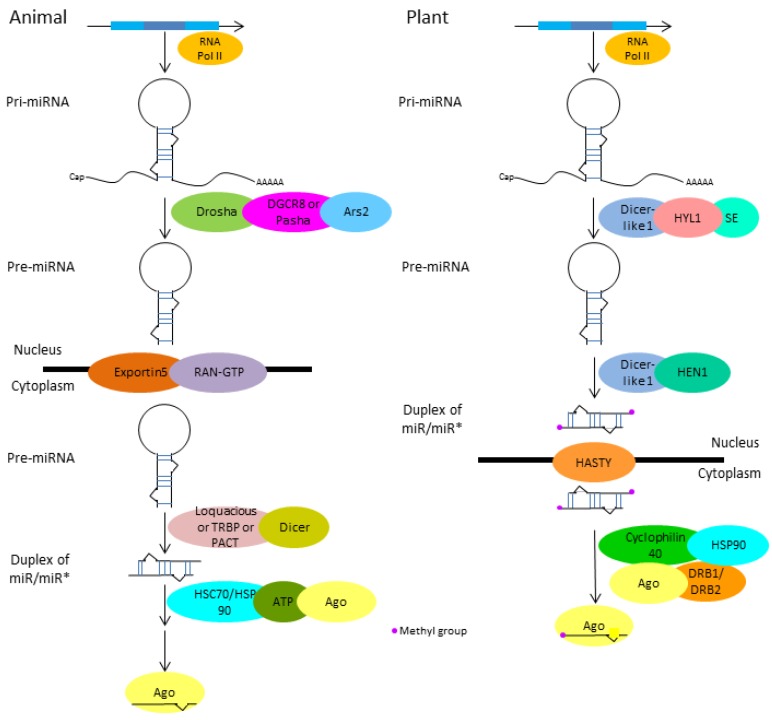
Comparison of the microRNA biogenesis pathways in animals and plants (summarized from [7,9,12,13,14,15,16,51,52,53,54]. In both plants and animals, the miRNA gene is transcribed into pri-miRNA (primary miRNA) with a 5’ Cap and 3’ polyA tail by RNA polymerase II, followed by processing into precursor-miRNA (pre-miRNA). However, different partners are involved in this process: Drosha, DGCR8 or Pasha, Ars2 in animals and Dicer-like1, HYL1, SE in plants. In animals, pre-miRNA is transported from the nucleus to the cytoplasm by Exportin5 and RAN-GTP for further processing by Loquacious or TRBP or PACT and Dicer to produce a duplex of miR/miR*. In plants, pre-miRNA is firstly processed by Dicer-like1 and then methylase HEN1 to produce the methylated duplex miR/miR* and then transported from the nucleus to the cytoplasm by HASTY. After that, selection and loading of miRNA to the RNA-induced silencing complex (RISC) is achieved through HSC70/HSP90, ATP, Ago in animals and Cyclophilin 40, HSP90, Ago, DRB1/DRB2 in plants. SE: Serrate, HYL1: Hyponastic leaves 1, HEN1: Hua enhancer 1, Ago: Argonaute protein, *: indicates passenger strand.

MiRNA systems in animals and plants are similar overall but have distinct features. Like protein coding genes, miRNAs in both animals and plants are transcribed as capped and polyadenylated primary miRNA (pri-miRNA) transcripts, followed by removal of the hairpin stem to form the precursor-miRNA (pre-miRNA ) and then further cleavage of the hairpin loop, resulting in the duplex of miR/miR* (* indicates passenger strand) [10,11,12,13]. Mature miRNAs are loaded on Argonaute (Ago) proteins to form the RNA-induced silencing complex (RISC) that binds to mRNA via target sequence complementarity to repress gene expression through inhibition of translation, degradation or decay of mRNA [6,10,14,15]. As mentioned above, the canonical biogenesis pathways between plants and insects are similar except for differences in cellular proteins and compartmentation (Figure 1).

There are three major routes of gene regulation mediated by miRNAs depending on the degree of sequence complementarity between miRNAs and their targets. These include mRNA degradation, repression of translation, and miRNA-mediated mRNA decay [6,16]. In general, perfect or almost perfect miRNA:target pairs will result in mRNA degradation, whereas imperfect pairs will lead to translational repression, which is more common in plants than animals [6]. Such miRNA-mediated translational repression is dependent on GW-repeat proteins in both plants and animals; for example, the SUO protein in plants [17] and GW182 proteins in animals act as the partner proteins to Argonaute (Ago) [18]). However, the dominant mode of post-transcriptional regulation by miRNAs differs between plants and animals. In some animals, such as the sea anemone *Nematostella vectensis*, cleavage of target mRNAs is mediated by nearly full complementarity between miRNAs and targets, similar to plants [19]. On the other hand, perfect miRNA:target pairing can result in repression of translation but not mRNA degradation in plants. For example, miR-172 regulates the expression of *APETALA2* via translational repression [20,21]. For plant phylogenetic outgroups, no *miRNA* gene is shared between green algae and land plants [22]. Nevertheless, in the green algae *Chlamydomonas*, the miRNA biogenesis pathway and properties are more in line with animals than plants. For example, the *Chlamydomonas* Dicer-like 3 (Dcl3) with a proline-rich domain is similar to the animal microprocessor Drosha [23]. It remains unclear whether these similarities reflect an established miRNA biogenesis and regulatory system in the last common ancestor of plants and animals which diversified into different lineages or resulted from convergent evolution.

## 2. Plant-Insect Interactions

Plants and insects interact in diverse and complex ways [24]. Plants need to defend themselves against destructive insect herbivory while attracting insect pollinators and beneficial insects. In response to herbivory by chewing or sap-sucking insects, plants have evolved constitutive and induced defense mechanisms to minimize tissue losses [25,26,27,28,29], while insects have evolved strategies to overcome such barriers [29,30]. In addition, plants must maintain positive interactions with some insect species, such as pollinators [31,32,33]. Therefore, plants have evolved strategies to deter herbivores yet attract these and other beneficial insects. At present, the involvement of miRNAs in these reciprocal interactions is in its infancy [34]. In the following sections, we explore evidence of miRNA regulating defense processes in the plants and offense strategies in the insects. We also discuss the scenario of plant-derived miRNAs that target insect physiological processes in both beneficial and pest species; conversely, the reciprocal scenario that miRNAs present in insect oral secretions could affect plant induced defenses is an intriguing question that lacks scientific support at present.

### 2.1. Plant miRNAs Targeting Plant Defense Responses

It is not surprising that miRNAs are important in regulating plant defense responses to insect herbivory. In melon, *Cucumis melo*, resistance to aphid herbivory in virus aphid transmission Vat^+^ lines is mediated by a two-fold mechanism both delaying phloem sap flow and targeting insect fecundity [35]. Susceptible Vat^−^ and resistant Vat^+^ near isogenic lines show distinct miRNA profiles in response to *Aphis gossypii* aphid attack [36], suggesting that not only are miRNAs involved in plant defense against aphid herbivory but that these differences contribute to plant resistance. Bioinformatic identification of putative miRNA targets includes genes that encode NAC transcription factors and proteins in phytohormone signaling and antioxidant pathways as well as senescence-associated (SAG) proteins [36]. Similarly, herbivory of the tea plant *Camellia sinensis* by caterpillars of the geometrid moth *Ectropis oblique* result in the differential regulation of over 150 miRNA species [37], supporting the involvement of miRNA in plant defense responses against insect herbivores.

Two important defenses in tobacco plants against caterpillar herbivory, nicotine and trypsin proteinase inhibitor, are constitutively present in the leaf but also highly induced in response to damage through jasmonate signaling [38,39]. In wild tobacco, *Nicotiana attenuata*, silencing of key proteins in the miRNA biogenesis pathway, such as RNA-directed RNA polymerase 1 (RdR1), Dicer-like 3 (Dcl3) or Dicer-like 4 (Dcl4), impair plant resistance against insect herbivory [40,41]. RdR1 is part of the processing machinery involved in small RNA biogenesis and transport and, as explained above, Dicer-like proteins are pivotal for mature miRNA processing ([42], Figure 1). In *N. attenuata*, RdR1 expression is induced either by treatment with the phytohormones jasmonic acid (JA) or salicylic acid (SA) or by application of caterpillar regurgitant but not by wounding itself. Silenced irdr1 plants are unable to defend themselves against caterpillars of the tobacco specialist *Manduca sexta* or the mirid bug *Tupiocoris notatus* and these plants suffer extensive damage when planted in their native habitat in the Great Basin Desert in Utah, USA [41]. This decrease in tobacco plant resistance reflects an impairment of nicotine biosynthesis rather than of volatile production [40]. Compared to wildtype *N. attenuata*, constitutive foliar nicotine levels are lower in dcl3- and dcl4-silenced plants and dcl3-silenced plants also have lower activity of the antinutritive trypsin inhibitor. Wounded tobacco leaves treated with caterpillar regurgitant elicit a strong jasmonate burst in the dcl3-silenced plants that mirror wildtype, while this reaction is slightly suppressed in dcl4-silenced plants. In response to wounding plus regurgitant or application of the phytohormones jasmonate-isoleucine or methyl jasmonate or *M. sexta* caterpillar infestation, nicotine levels slightly increase in the dcl3-silenced line, but do not attain the reaching wildtype levels, and this phenomenon does not occur in dcl4 line. These studies highlight the importance of miRNA in the regulation of plant defensive responses against insect herbivores [40].

### 2.2. Insect miRNAs Targeting Plant Defense Responses

During feeding, insect herbivores encounter a diverse array of noxious and/or toxic specialized plant metabolites [43]. Insect herbivores mitigate the potentially dangerous effects of these compounds by either moving to a different plant source or detoxifying the compounds with enzymes including cytochrome P_450_s (CYP), esterases, or glutathione *S*-transferases (GST) [44,45,46,47,48,49]. Recent studies have shown that these detoxification enzymes may be regulated by endogenous miRNAs, particularly the CYPs, as part of the insect offenses to plant defenses. Silencing of the endonuclease Dicer-1 and of the core component of the RISC complex Ago-1 increases the sensitivity of cotton aphids (*A. gossypii*), adults to plant-derived polyphenolic compounds, such as gossypol and tannic acid [50]. Gossypol, produced in stem, leaves, flower buds and seeds of cotton, generates its strong anti-herbivory effect by acting as a general inhibitor of dehydrogenases [26]. Tannic acid is more ubiquitous and found in diverse plant species. The role of tannic acid is less well understood but possibly acts as an antinutritive agent [28]. The silencing of *dicer-1* or *argo-1* in *A. gossypii* results in higher susceptibility of aphids to dietary tannic acid and gossypol or tannic acid only, respectively.

Ma and colleagues [55] identified differentially expressed aphid miRNAs in response to plant allelochemicals and they bioinformatically predicted that a subset of them may regulate genes encoding proteins involved in xenobiotic detoxification. To prove the concept, they further showed that miR656a-3p binds to the 3’-UTR of the transcript encoding the putative detoxification enzyme CYP6J1, promoting its degradation; expression of miR656a-3p is low when aphids are fed on a diet containing 2-tridecanone, tannic acid or quercetin that may result in higher CYP6J1 levels that may be involved in the detoxification of these compounds, though these final steps were not shown. Adult aphids of the tobacco specialist *Myzus persicae* own two miRNA species, let-7 and miR-100, that are predicted to negatively regulate transcript levels of *Cyp6Cy3* encoding a nicotine detoxification enzyme [56]. The nicotine-tolerant *M. persicae* subspecies *nicotianae* has lower endogenous levels of these miRNAs and is thus more able to cope with nicotine. Feeding with miRNA mimics or inhibitors added to an artificial diet makes *M. persicae nicotianae* aphids more or less sensitive to the toxic effects of nicotine, respectively [56].

Insects often use similar strategies to detoxify plant specialized metabolites or insecticides [44,45,46,47,48,49,57], therefore, as expected, miRNAs are also implicated in pesticide resistance. Caterpillars of the diamondback moth, *Plutella xylostella*, are highly destructive pests of Brassica crops [58]. Several miRNA species that regulate resistance to the ryanoid insecticide chlorantraniliprole were identified in this insect [59]. miR-2b-3p and miR-14b-5p post-translationally suppress two *cytochrome P_450_ monooxygenase* genes, *CYP9F2* and *CYP307a1*, respectively, which are most likely involved in insecticide detoxification [59]. A diet containing miR-2b-3p mimics provided to a *P. xylostella* line resistant to the pyrethroid insecticide deltamethrin results in greater lethality, presumably because miRNA-mediated suppression of cytochrome P_450_ enzyme decreases the insect ability to detoxify the pesticide.

Another strategy adopted by insects to avoid the detrimental effects of pesticides is through target upregulation or insensitivity. Acetyl-CoA carboxylase (ACC), a key enzyme in the fatty acid biosynthesis, is the target of the insecticide spirotetramat [60]. miR276 and miR3016 post-transcriptionally regulate *ACC* transcript quantity; the quantity of these miRNAs is lower in the spirotetramat-resistant strain of the cotton aphid *A. gossypii* [61]. Alteration of miR-276 or miR-3016 levels in the aphid thus affects *ACC* transcript levels and insecticide susceptibility [61].

Field and laboratory strains of the devastating pest, the European corn borer (ECB), *Ostrinia nubilalis*, have evolved resistance against transgenic corn containing the *Bacillus thuringiensis* (Bt) insecticidal crystalline (Cry) protein [62]. Comparison of miRNA profile after ECB larvae fed on Bt corn revealed 35 miRNAs that are differentially expressed between susceptible and resistant larval strains, with a subset of these bioinformatically predicted to target Bt-resistance genes, such as *cadherin* and *aminopeptidase N* [63,64]. In Cry-resistant ECB, miRNA targeting *aminopeptidase N (APN*) genes are upregulated, which may result in lower APN levels. In contrast, miRNA *onu-novel-29* targeting the *cadherin* gene is downregulated potentially resulting in higher levels of this potent Cry-binding protein. In addition, ABC transporters are putative targets of these miRNA. Nevertheless, these observations still need to be supported by empirical data.

Changes in endogenous miRNAs of the insect may also affect endocrinological targets [65]. *A. gossypii* fed on near-isogenic Vat^+^ or Vat^–^ lines for two days exhibited a differential miRNA profile [66]: there is a trend of less miRNA species in aphids fed on the resistant Vat^+^ melon. Bioinformatic prediction of putative targets in these aphids revealed that these miRNAs may be associated with the regulation of genes involved in development and reproduction [66]. Therefore, the reduced levels of these miRNAs may contribute to the observed lower growth and fitness of aphids fed on the resistant Vat^+^ melon [66]. Collectively, these studies highlight the role of miRNAs to regulate insect detoxification mechanisms and the potential of these as targets in biotechnological approaches addressed to plant protection.

### 2.3. Plant miRNA Targeting Herbivorous Insects

Firstly, if plants produced miRNAs to interfere with insect herbivores, it would need to access insect tissues. For foliar or root feeding insects, release of cellular contents during tissue ingestion may trigger delivery of plant-derived miRNAs. For phloem-feeders, miRNAs must be present in the viscous sap. In plants, miRNA sorting governs whether miRNAs are secreted extracellularly or remain within the producing cell [67]. Dicer enzymes differ in how they process the pri-miRNA to generate different miRNAs with different functions [12]. For example, Dcl1 processes pri-miRNA to a 21-nt miRNA with a U residue at the 5’-end that mediates post-transcriptional gene silencing. Dcl3 processes pri-miRNA to generate a 24-nt miRNA with an A residue at the 5’-end and UCA residues at the 3’-end that is loaded onto Ago4 to possibly drive transcriptional gene silencing through cytosine methylation of miRNA genes. Processing of pri-miRNA by Dcl may also generate a 21-nt miRNA with an A residue at the 5’-end that is then loaded onto Ago2, a protein that can be secreted extracellularly [68]. Whether this type of mechanism plays a role in the release of miRNAs into phloem sap remains unknown.

**Figure 2:**
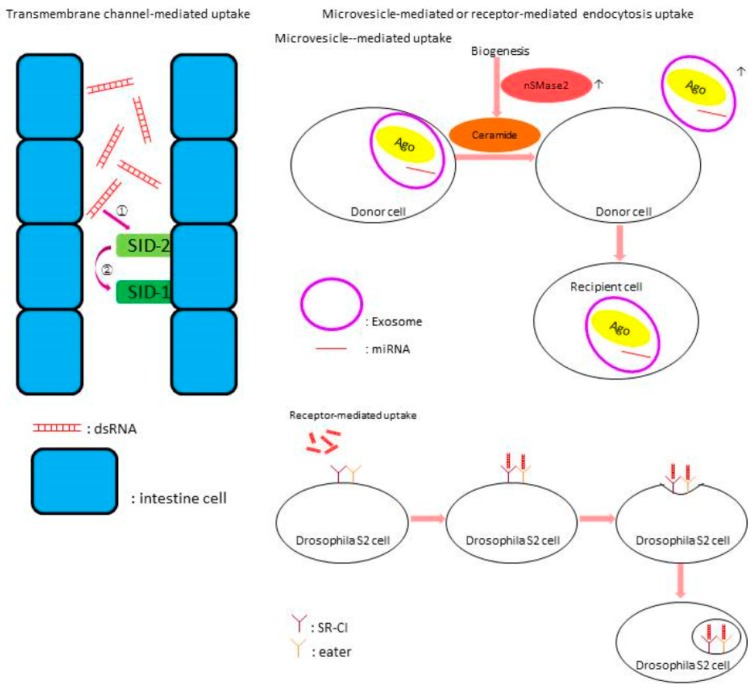
Potential mechanisms of miRNA interactions between plants and insect herbivores. Proposed interactions are based on current understanding of other organisms (summarized from [72,73,77,78]. The left panel illustrates the transmembrane channel-mediated pathway in *C. elegans*. Double-stranded RNA (dsRNA) binds to SID-2 (①) then SID-1 (②) to initiate endocytosis. The right panel illustrates microvesicle-mediated (top) and receptor-mediated (bottom) pathways. In the microvesicle-mediated pathway, exosomes-containing RNAs and components of RISC-like Ago are secreted by cells through ceramide-dependent secretory machinery. In addition, neutral sphingomyelinase 2 (nSMase2), a key regulatory enzyme in ceramide biosynthesis, regulates ceramide production and hence the amounts of secreted miRNA. In the receptor-mediated pathway present in *Drosophila* S2 cells, binding of dsRNA to two receptors, SR-CI and eater, results in the internalization via receptor-mediated endocytosis.

Once secreted, the miRNA must then be taken up by cells in the other organism. Prior to the discovery of miRNAs, double-stranded RNAs (dsRNA) were shown to be transferred from one organism to another, either by transmembrane channel-mediated mechanisms based on RNA transporter-like proteins or microvesicle-mediated or receptor-mediated endocytosis [69,70,71,72] (Figure 2). These mechanisms have recently been elucidated in the nematode *C. elegans*. In the *C. elegans* gut, the transmembrane protein SID-2 binds to the long dsRNA followed by interaction with SID-1 leading to endocytosis [73]. However, there are SID-1 independent pathways where dsRNAs silence the expression of target genes through RNA interference (RNAi) [74,75]. Another mechanism of RNA delivery involves extracellular vesicles [76]. Microvesicles, which can be vesicles, exosomes, or apoptotic bodies, are carriers transporting and protecting the miRNAs from degradation [71]. Components of the RISC are contained within these vesicles to ensure miRNA bioactivity [77]. In mammals, the enzyme neutral sphingomyelinase 2 (nSMase2) promotes the release of miRNAs from microvesicles [78]. These proteins, however, do not appear to have orthologs in insects. In the fruit fly, *Drosophila melanogaster*, two scavenger receptors, SR-CI and Eater, are involved in the internalization of dsRNAs, suggesting that receptor-mediated endocytosis may also be involved in miRNA transport [72].

Thus, there are several mechanisms to facilitate miRNA transfer within and between organisms and host plant-derived miRNAs have been identified in herbivorous insects. Two aphid species, the green bug *Schizaphis graminum* and the yellow sugarcane aphid *Sipha flava*, were fed on sorghum or barley plants [79]. By using next generation sequencing, 13 and three miRNAs were identified from sorghum and barley samples, respectively, in whole bodies of these aphids. This indicates that host plant miRNAs can be taken up in the insect, at least in the case of hemipteran aphids [79]. Plant-derived miRNAs were detected in the aphid *A. gossypii* tissues after feeding on melon phloem sap [66]. It is of interest that miRNAs were also detected in the honeydew when aphids were fed with radiolabelled miRNAs [80]. Plant-derived miRNAs were also found in haemolymph and tissues of silkworm, *Bombyx mori*, caterpillars fed on mulberry (*Morus*) leaves [81], indicating that miRNAs from the plant are also able to traverse from the gut into caterpillar tissues.

To date, as shown above, a few studies have identified plant-derived miRNAs in insects and putative targets have been bioinformatically predicted. The next steps are to experimentally confirm these exciting observations to further understand the implications of these close ecological interactions. *A. gossypii* aphids fed on either resistant Vat^+^ or susceptible Vat^−^ melon lines and the plant-derived miRNA in the aphids showed unique qualitative and quantitative profiles; only two compared to five plant miRNA families were identified in aphids fed Vat^+^ vs. Vat^−^ melon lines, but the miR166 family was more abundant in Vat^+^. Silkworm, *Bombyx mori*, caterpillars feeding on mulberry leaves rapidly accumulates the plant-derived miRNA miR-166b in its haemolymph and fat body, with highest levels observed within 30 min of feeding [81]. However, a physiological target for this miRNA was not identified.

The ability of plant host-derived miRNAs to affect insect physiology remains controversial. In several plant-coleopteran (beetle) systems, for example in the maize-western corn rootworm and tomato-Colorado potato beetle systems, longer host derived dsRNAs but not miRNAs are taken up and processed by the insect machinery [82,83]. In contrast, in the plant-lepidopteran (moths and butterfly) systems tested, caterpillars remain recalcitrant to the effects of either longer dsRNA or miRNA. This may reflect the harsher environment of the lepidopteran gut where the advanced peritrophic membrane may limit miRNA availability and the alkaline environment is highly destructive to RNA [84,85,86].

### 2.4. Insect Subversion

Plant defense responses against insect herbivory are fine-tuned through distinct phytohormone signaling pathways that interact to produce an outcome that specifically targets the herbivore [87]. The jasmonate (JA) signaling cascade is a key pathway activated in response to chewing insects, such as caterpillars or beetles, and the outcome of this cascade is modulated by other hormones, such as ethylene, salicylic acid, gibberellins, to name a few. However, effectors in caterpillar regurgitant modify plant responses to herbivory, in either negative or positive manners, primarily by influencing defense signaling through phytohormone crosstalk [88]. In the tobacco silenced RdR1 (irdr1) lines, artificial damage and treatment with caterpillar regurgitant respectively result in lower JA and higher ethylene levels at 30 and 300 min, after wounding compared to wildtype [89]. Since JA-induced nicotine biosynthesis is attenuated by ethylene in *N. attenuata*, these results suggest that caterpillar regurgitant leads to modulation of plant miRNAs to suppress nicotine biosynthesis [90]. Further identification of *N. attenuata* miRNA populations in wounded compared to regurgitant-treated or control plants revealed seven wound-induced and 12 regurgitant-specific plant miRNAs [91]; of these 10 miRNAs were regurgitant-specific and JA-independent, six miRNAs were regurgitant-specific and inhibited by JA and three miRNAs were regurgitant-specific and JA-dependent. Bioinformatic prediction of miRNA targets further suggest that miR390 in particular, which is a regurgitant-specific and JA-inhibited miRNA, could negatively regulate trans-acting siRNA (tasi-RNA), which results in the inhibition of root growth and accumulation of the defensive specialized metabolite nicotine.

### 2.5. Plant miRNA: Interactions with Beneficial Insects

Plant-insect interactions are multi-trophic and entail many different players. For instance, the interaction between plants and insects may be affected by viral or bacterial pathogens (see below). Tomato plants (*Solanaum lycopersicum*) infected with the cucumber mosaic virus (CMV) produce a more attractive volatile profile to bumble bees, *Bombus terrestris*, than healthy plants [92]. It is hypothesized that even though tomatoes are self-pollinating, this phenomenon serves to increase pollination frequency in an unhealthy plant [92]. *Arabidopsis thaliana* mutants were used to tease out the responsible party: pathogen-derived short interfering RNAs (si-RNA) versus plant-derived miRNAs. Although results obtained so far implicate the plant-derived miRNAs, further research needs to be performed to confirm these preliminary observations in tomatoes.

Though contentious, plant-derived miRNAs may be involved in plant-pollinator interactions, particularly in caste differentiation. The differentiation of female bees into different castes, workers or queen, reflects complex genetic and environmental interactions [93]. Nutritional diet is a key trigger of this process: larvae fed with royal jelly regurgitated by nurse bees differentiate into large, reproductive queens, whereas larvae fed with a mixture of pollen and honey, known as bee bread, differentiate into smaller, sterile workers responsible for hive maintenance and foraging [94,95]. The role of pollen-derived miRNAs in regulating caste differentiation has been queried. In honey bees, *Apis mellifera*, plant pollen miR-156a is abundant in midguts in worker or nurse bees, where it is associated with the undigested bolus despite being at lower levels in other tissues [96]. This suggests that plant miR156a has been excreted. In another study, bee larvae fed with a diet containing a pool of 16 pollen-associated miRNAs developed into smaller adults with reduced reproductive capacity, reminiscent of worker bees. Consistently, pollen-derived miR-162a negatively regulates the bee *AmTOR* gene that promotes differentiation into the worker caste [95], supporting the idea that plant-derived miRNAs may play a role in the regulation of this highly specific process.

## 3. Biotechnological Applications

Given the role of miRNAs as master regulators of critical physiological processes in insects, using dsRNAi or miRNA mimics in transgenic plants to target insect genes has many potential, exciting applications in agriculture. Present day agriculture is faced with problems concerning current pest management practices, including increased pesticide resistance, environmental pollution, and toxic effects on non-target organisms, including humans [97,98]. In addition, insect devastations are predicted to worsen under future changing climatic conditions [99]. Thus, an approach that targets miRNAs in insects using transgenic plants expressing anti-miRNAs or miRNA mimics is a promising avenue to pursue. The clear advantage is that miRNAs have the potential to target multiple genes in pathways regulating development, growth, reproduction, and detoxification to achieve sustainable pest management [100].

For insect herbivores, genes expressed in the insect midgut cells are important targets that have the potential to disrupt feeding or detoxification of plant specialized metabolites or insecticides. Therefore, in this environment, one major concern is the degradation of dsRNA by the gut pH or nucleases present in the gut. Thus, strategies to provide a continuous supply of dsRNA or using vesicles to carry the dsRNA to the desired target cell are current areas of research [78,101]. Disruption of nucleases by dsRNAi has been a successful strategy to optimize dsRNAi approaches in planta; whitefly *Bemisia tabaci* mortality is increased to 50% when stacked dsRNAi constructs are used to target the genes encoding sucrase1 (SUC1), AQP1 aquaporin as well as two nucleases (dsRNase1 and dsRNase2) [102].

Another consideration for the implementation of dsRNAi technology is the accumulation in the proper tissue or resource that the insect feeds on. For example, for phloem-feeding hemipterans, plant sap must contain the dsRNA vector compared to foliar or root tissue that is more appropriate for lepidopteran larvae or coleopteran larvae and adults [101]. Lastly, target specificity is critical. There has been success with dsRNAi designed against *vacuolar-type proton ATPase* (*vATPase*) transcripts in many insect genera targeting and causing specific lethality in fruit flies (*Drosophila* sp.) or flour beetles (*Tribolium* or *Tenebrio* sp.) or pea aphids (*A. pisum*) or tobacco hornworms (*M. sexta*) [98]. In fact, careful design can even generate species-specificity. Four *Drosophila* species were selectively killed by dsRNAi targeting the 3’UTR of their *tubulin* gene [98].

The next step is to generate transgenic plants using artificial miRNA backbones (amiRNA) or dsRNAi to target specific insect physiological and endocrinological processes. In insects, the polymer chitin present in the insect cuticle is degraded by the enzyme chitinase during insect molting [103]. In *Helicoverpa armigera* caterpillars, miR-24 targets the 3’-UTR of the chitinase gene resulting in suppression by either transcript degradation or translational repression [104]. Transgenic tobacco expressing miR-24 fed to *H. armigera* caterpillars delays molting and enhances lethality within 2 days. In another example, acetylcholinesterase acts at cholinergic synapses to hydrolyze the excitatory neurotransmitter acetylcholine [105]. Transgenic plants targeting the *acetylcholinesterase 2* transcript of the aphid *M. persicae* using an artificial miRNA show stronger resistance than plants expressing the same gene within an artificial virus-specific hairpin vector (hpRNAs) [106].

These agribiotechnological approaches for pest control can be tailored to target the expression of multiple genes, affecting the same or distinct physiological processes. The enzyme cytochrome P_450_ 307a1 (Cyp307a1) is involved in the biosynthesis of insect steroidal ecdysteroids that bind to the ecdysone receptor (EcR) to initiate and regulate the developmental process of molting [107]. In the rice stem borer, *Chilo suppressalis*, miR-14 post-transcriptionally targets the genes that encode both Cyp307a1 and EcR [108]. Rice plants that constitutively express miR-14 show enhanced resistance against the rice stem borer. As shown in the section on insect miRNAs targeting offensive responses, miRNAs are involved in the regulation of insect mechanisms to detoxify plant allelochemicals and insecticides. Therefore, the exploitation of miRNA-based strategies (mimics, inhibitors etc.) in the host plant to target these detoxification enzymes provides a potentially excellent pest control system.

Although these studies highlight the advantage of targeting miRNA pathways for insect control, several obstacles remain for the optimization of this approach. These include insufficient delivery that may reflect relatively poor miRNA stability and off-target effects [2]. To reduce miRNA stability issues, RNA chemical modifications, such as phosphorothioate backbone modification, ribose 2’-hydroxyl group modification and locked or unlocked nucleic acids, have been investigated for use in the development of synthetic miRNAs [2]; however, these adaptations may not be applicable for use in transgenic plants. Additionally, novel delivery methods, such as through viral vectors and non-viral vectors including lipid-based or polymer-based vectors, nanoparticles, as well as endogenous vesicles such as exosomes, are active areas of research [2].

In conclusion, plants and insects have already taken advantage of miRNA regulation of physiological processes to manipulate each other. In both groups of organisms, miRNA regulation of key physiological processes makes these signal molecules an attractive target for biotechnological approaches addressed to crop protection. However, it is of the utmost importance to understand the nature and underlying mechanisms controlling miRNA-mediated plant-insect interactions. Currently, dsRNAi applications can also be improved to make this process more efficient by targeting endogenous degradative machinery, such as nucleases, in addition to genes that encode proteins of key physiological relevance. In addition, potential effects on non-target beneficial organisms must be considered carefully. Indeed, whether or not plant-derived miRNAs were taken up by insects, there would be the possibility of affecting non-target species, including beneficial insects or herbivores and omnivores that could ingest the plant material, including humans. These concerns must always be taken into account and sufficient testing conducted to ensure the absence of undesired effects.
